# Psychosocial interventions for improving marital intimacy, sexual satisfaction, and quality of life of women and couples with infertility problems in low and middle-income countries: Systematic review protocol

**DOI:** 10.1371/journal.pone.0335068

**Published:** 2025-10-30

**Authors:** Stella Sarpomaa Oppong, Florence Naab, Isaiah Osei Duah, Samuel Ankamah, David Owiredu, Francisca Happy Ametor, Mercy Monde Imakando, Josephine Mpomaa Kyei, Anthony Danso-Appiah

**Affiliations:** 1 School of Nursing and Midwifery, College of Health Sciences, University of Ghana, Accra, Ghana; 2 Department of Optometry and Visual Science, College of Science, Kwame Nkrumah University of Science and Technology, Kumasi, Ghana; 3 Department of Biological Sciences, College of Science, Purdue University, West Lafayette, Indiana, United States of America; 4 University of Ghana Library System, University of Ghana, Accra, Ghana; 5 Department of Epidemiology and Disease Control, School of Public Health, University of Ghana, Accra, Ghana; 6 Centre for Evidence Synthesis and Policy, University of Ghana, Accra, Ghana; 7 Department of Population, Family and Reproductive Health, School of Public Health, University of Ghana, Accra, Ghana; 8 Department of Obstetrics and Gynaecology, Women and Newborn Hospital, University Teaching Hospitals, Lusaka, Zambia; 9 Africa Communities of Evidence Synthesis and Translation (ACEST), Accra, Ghana; Adolescent Health Champions, UNITED STATES OF AMERICA

## Abstract

**Introduction:**

Psychosocial interventions can be effective in improving health outcomes and quality of life of persons with infertility problems. This systematic review aims to document available psychosocial interventions for infertility-related problems in Low- and Middle-Income Countries (LMICs) and to assess their effectiveness for marital intimacy, sexual satisfaction, and quality of life (QoL) in people with infertility problems.

**Methods:**

Studies will be considered eligible if they are randomized controlled trials (RCTs), quasi-experimental studies, and Observational studies (cohort, case-control, cross-sectional studies) involving women and couples with infertility problems and living in a LMIC who received a psychosocial intervention to improve marital intimacy, sexual satisfaction, and QoL. We will search PubMed, SCOPUS, CINAHL, LILACS, CENTRAL, and PsycINFO (EBSCO) from inception to 31^st^ March 2025, without language restriction, using all the relevant search terms and their synonyms, singular and plural forms and British and American spellings, together with the individual countries according to the Sheffield Centre for Health and Related Research (ScHARR) 2022 classification of LMICs. We will also search conference proceedings, preprint repositories, dissertation databases, the World Health Organisation, and government databases for additional studies. We will contact trial registries and experts for unpublished trials and hand-search reference lists of retrieved papers for studies missed by our searches. The retrieved studies will be exported to Rayyan for de-duplication and study selection using a pre-tested study selection flowchart developed from the study eligibility criteria. At least two reviewers will independently select studies, extract data with pretested data extraction sheet, and assess the quality of the included studies using validated tools. Dichotomous data will be assessed and reported as odds ratio (OR) or risk ratio (RR), and for continuous outcomes, mean difference (MD) will be used; all will be reported with their 95% confidence interval (CI). Heterogeneity will be assessed graphically by inspecting overlapping CIs and quantitatively using the I^2^ statistic. Sensitivity analysis will be performed to test the robustness of the pooled estimates if the data permit, and the overall quality of the evidence will be assessed using the GRADE approach.

**Expected outcomes:**

This systematic review and meta-analysis will identify effective psychological interventions and elaborate on the specific components to optimize and improve the psychosocial well-being of persons with infertility experiencing problems with sexual satisfaction and intimate relationships. The review will also uncover previously misclassified effective interventions due to limited data and small sample sizes from underpowered primary studies. Highlighting the existing evidence on available psychosocial interventions will inform clinical application, public education, and counselling services to optimize QoL of infertile couples and, importantly, inform the design of further studies to interrogate the region-specific application of these interventions.

**Dissemination and protocol registration:**

The findings of this study will be disseminated through stakeholder forums, conferences, and peer-reviewed publications. The review protocol has been registered in the International Prospective Register for Systematic Reviews (PROSPERO)- CRD CRD42023480729.

## Introduction

Infertility remains a public health challenge due to its negative impact on reproductive ─ sexual satisfaction [1,2] and marital intimacy [3,4]; cognitive ─ anxiety [4,5], depression [6,7], and psychological distress [8]; social functioning, i.e., social relationships [9–11], and overall QoL [12,13]. Half of the women with infertility in LMICs experience lifetime abuse that culminates in anxiety and depressive episodes [6,14,15]. Psychosocial interventions may offer positive coping mechanisms and resilience to ameliorate the prevailing psychological distress among infertile couples [16–18]. Psychosocial interventions are mainly accessible to women seeking Assisted Reproductive Technology (ART) [16,19–21] and not all individuals with infertility problems [22]. Globally, over 50 million cases are indicated for ART. Yet, only a small fraction in LMICs receive this treatment, particularly in SSA, due to limited access and high treatment costs [23–25]. Given the paucity of data, it is unclear, and therefore largely unknown, which factors are the main drivers of ART uptake.

As a social construct, the discussion of infertility is of paramount importance in LMIC communities, as it is closely linked to procreation and lineage [22,26]. Hence, the inability to fulfill expectations of procreation as couples infringe on their social and psychological well-being. Worryingly, a number of infertile women and couples are not accorded the due respect and recognition in society [22,26]. They are denied many privileges in the family and are seen as worthless and useless [27,28]. These prevailing stressors reduce their psychological resilience, which in turn affects their social relationships [9], marital intimacy [3], and sexual functions [29].

Infertility treatment is disproportionately underutilized in LMICs [30,31] due to the high cost of treatment and the limited financial resources of those affected. The lack of access indirectly causes significant emotional distress [32,33]. Systematic reviews have shown that individuals with infertility problems in LMICs are less likely to have access to any form of ART [31,34], even routine medical care, including psychosocial interventions [35]. While psychosocial interventions are conceptualized in high-income countries and incorporated in the health systems, LMICs are yet to incorporate them in the management of infertility [31].

### How psychosocial interventions might work?

Psychosocial interventions do not address the underlying biological cause of infertility, but can improve healthy emotions, attitudes, and habits that ultimately enhance the QoL of couples and/or partners with infertility problems [10]. The mechanisms through which psychosocial interventions operate is not well understood but they target the cognitive, emotional, and relational processes, leading to desired changes in individuals’ mental health and functional outcomes [36]. The interventions grouped according to mode of delivery and inherent psychometric properties, such as counselling, psychodynamic therapy, cognitive behavioural therapy (CBT), mindfulness-based stress reduction (MBSR), humanistic therapy education, behavioural change techniques, yoga, and others, are widely recognized as effective for various psychosocial problems [37–40].

For example, counselling allows couples to process feelings of depression, anxiety and grief whilst being provided with access to emotional support from health professionals and experienced peers. Together, this creates a safe environment that allows individuals to express their concerns about problems they are going through, in this case infertility-related psychosocial problems [39]. In psychodynamic therapy, the focus is on uncovering unconscious thoughts and feelings, facilitating insight into emotional struggles through techniques like free association and dream analysis [40]. This process is complemented by the exploration of transference, where clients project past relational dynamics onto the therapist, allowing them to better understand their emotional responses [41]. Cognitive-behavioural therapy emphasizes cognitive restructuring, which involves identifying irrational thoughts and negative ideations and instead allows substitution with positive reinforcement coping strategies to meet conceived expectations [42]. It also incoporates behavioural activation, which encourages engagement in positive activities and thoughts that enhance emotional regulation and coping skills [43]. Adopting mechanisms such as cognitive restructuring in CBT that focuses on challenging cognitive distortions and fostering more adaptive thought patterns have been proved to be very effective [43]. Mindfulness-based stress reduction incorporates mindfulness meditation and yoga that helps individuals to manage daily stress and emotional pain, and builds resilence [44]. By fostering greater awareness and acceptance of one’s emotional state, MBSR can enhance coping skills for individuals facing infertility challenges [45].

Humanistic therapy education, however, prioritizes the therapeutic alliance by building a trusting relationship that fosters self-exploration, empowerment, and personal growth [46], whereas behavioural change techniques, such as exposure therapy and skill acquisition, help clients confront their fears and develop coping strategies [47]. Other interventions such as yoga aims to encourage the individual to focus on the present condition, and by so doing, it helps to alleviate anxiety [48]. This may help modulate infertile couples expectations following treatment and consequently alleviate the self-generated ideation of hopelessness [49]. Couples therapy focuses on addressing relationship issues, helping partners to navigate the emotional challenges of infertility together [50] and in assessing therapy sessions together tends to enhance couples ability to effectively communicate, resolve conflicts, and importantly develop shared coping strategies to improve emotional resilience and interpersonal relationships [50].

Central to the effectiveness of psychosocial interventions is the therapeutic alliance, characterized by trust, empathy, and open communication, which significantly enhance clients’ engagement and motivation [51]. Emotional processing further plays a crucial role, enabling clients to identify, express, and understand their emotions, ultimately promoting healing [52]. Additionally, self-awareness and insight gained through reflective exploration allow clients to integrate new-found understandings into their daily lives, facilitating lasting personal growth and improved mental health outcomes [53]. Understanding these mechanisms equips therapists to tailor their approaches to the needs of the client to enhance the effectiveness of the psychosocial intervention. Given the mechanisms through which psychosocial interventions work—such as improving communication, building trust, fostering amicable resolution of indifferences, and enhancing emotional bonds, these can improve marital intimacy, sexual satisfaction, and QoL of couples or individuals with infertility.

### Rationale for this systematic review

Couples or persons with infertility problems are predisposed to societal stigma that negatively affects their psychological wellbeing. The decline in psychological resilience from infertility-related stress and events affects the social relationships, marital and sexual functions of individuals living with infertility [29]. Accumulated evidence, though fragmented, suggests an urgent need to manage infertility beyond medical care [54]. Adoption of psychosocial interventions may remediate psychological stress associated with the diagnosis, and management, of infertility [16,55–61].

Addressing infertility is an integral part of sexual and reproductive health and rights [62]. However, in many countries, policies and services on infertility are inadequate. Tackling infertility and its negative psychosocial impact is essential for the achievement of Sustainable Development Goal (SDG) 3 [63], which aims to ensure healthy lives and well-being for everyone at all ages, and SDG 5, which seeks to attain gender equality and empower all women and girls. Furthermore, providing psychosocial interventions for infertility is fundamental for realizing the human rights related to the highest attainable standard of physical and mental health and the ability to make decisions [64]. This is likely to enhance their self-esteem, and, likely to improve their participation in leadership roles, and potentially improve their decision-making about infertility [65]. Some primary studies have investigated the role of psychosocial interventions on marital intimacy and sexual satisfaction, but the results are fragmented and inconclusive [66,67]. Therefore, there is a need for a systematic review to collate existing studies and obtain a pooled estimate of the effectiveness of psychosocial interventions for persons with infertility problems.

To ensure this systematic review is not duplicating existing reviews, searches were conducted in relevant databases, and three existing systematic reviews that investigated psychosocial interventions among infertile couples were retrieved [66–68]. One of these systematic reviews investigated anxiety and depression [68], another, somewhat outdated review, investigated only sexual satisfaction [67], and the last review focused on the Middle East context [66]. None of the earlier systematic reviews was as comprehensive as the current systematic review. This review aims to assess the effect of psychosocial interventions in improving sexual satisfaction, marital satisfaction, marital intimacy, sleep quality and infertility-related QoL. It also seeks to explore (psychometric) characteristics of available psychosocial interventions including feasibility in resource-limited settings, adaptability to other LMICs settings, cost of the intervention. Additionally, the review examines the level of expertise required to deliver the intervention, whether the intervention can be delivered in peripheral health facilities by non-specialist healthcare providers and acceptability by patients. 

A comprehensive synthesis of studies on psychosocial interventions for infertility within LMICs will help to objectively and rationally uncover the strengths and limitations in existing psychosocial interventions. The characteristics of existing interventions and the contexts in which they were administered need to be explored for adaptability and feasibility in sub-Saharan African countries. This is expected to help conceptualize care and improve marital intimacy, sexual satisfaction, and QoL of individuals with infertility in LMICs settings as recommended by the World Health Organization (WHO) [69]. The main objective of this systematic review is to systematically synthesize existing data captured from the literature and other sources and provide robust evidence on the availability and effectiveness of psychosocial interventions to improve the well-being of people with infertility problems in LMICs. Specifically, the study aims to: 1) assess the effectiveness of documented psychosocial interventions for improving QoL, marital intimacy, and sexual satisfaction among individuals living in LMICs going through infertility problems, 2) assess the quality of life of individuals living in LMICs going through infertility, 3) assess (psychometric) characteristics of the documented psychosocial interventions in LMICs, and 4) assess psychological concerns of people going through infertility problems in LMICs.

## Methods

This protocol will follow the standard guidelines specified in the Cochrane Handbook [70], and will be reported according to the Preferred Reporting Items for Systematic Review and Meta-Analysis protocol (PRISMA-P) guidelines for transparency and rigor [71,72] (S1 Table). The full review will be reported using the PRISMA guidelines [72]. The review will use comprehensive search methods that will attempt to retrieve all possible studies that meet pre-specified eligibility criteria and will use explicit, transparent, and methodical processes to select and appraise studies, extract, and analyse data, and use the PRISMA flow diagram [71] (S1 Fig) to delineate the methodological flow of studies from retrieval to analysis.

### Patient and public involvement

The review questions and outcome measures have been developed in collaboration with the relevant patient and consumer involvement and are informed by their priorities, experiences, and preferences in line with the GRIPP2 reporting checklists [73]. The results of the review will be shared with the relevant wider client communities who will also be involved in the dissemination of the results.

## Criteria for considering studies for review

### Types of studies

Randomized controlled trials (RCTs), quasi-RCTs, and Observational studies (cohort, case-control cross-sectional studies) reporting on any psychosocial intervention used to improve QoL, marital intimacy, and sexual satisfaction in people with infertility problems living in LMICs as defined by ScHARR [77], will be eligible for inclusion. Reviews will not be considered for inclusion; however, we will snowball review papers for any potentially primary eligible studies missed in our primary searches and consider them for inclusion. Where the study reports a country or regional estimate without a well-defined sample (a representative sample or sub-sample of the source population) or data from secondary analyses, it will not be eligible for inclusion. Expert opinions, commentaries, newsletters, case series, and case studies will be excluded. Studies that assessed psychosocial problems such as depression, anxiety, stress, self-esteem but did not investigate sexual satisfaction, marital intimacy and, fertility-related QoL will be excluded.

### Participants

Individuals (women or couples) with infertility problems living in a LMIC who received psychosocial intervention aimed to improve their QoL, marital intimacy, marital satisfaction, and sexual satisfaction. Infertility is defined by the World Health Organization (WHO) as a disorder of the male or female reproductive system that leads to failure to achieve a pregnancy after 12 months or more of regular unprotected sexual intercourse [74]. This systematic review will include women and couples diagnosed with primary infertility where a couple has never been able to conceive a pregnancy after a minimum of 12 months of attempting to do so through unprotected intercourse. It will also include those with secondary infertility, characterized as the inability to conceive or carry a baby to term after 12 months of unprotected intercourse, in a woman who has previously had a baby without fertility treatments. Participants must have received any form of psychosocial intervention that sought to improve their marital intimacy, marital and sexual satisfaction, and overall fertility-related QoL.

Studies focusing exclusively on men with infertility issues will not be eligible for inclusion.

### Intervention

Psychosocial interventions, may include support programs, counseling, CBT, educational interventions, acceptance and commitment therapy (ACT), and relaxation techniques. Interventions may be women, couples, group, or Internet-based. Interventions will be stratified by format, frequency, duration, delivery mode, setting, provider qualifications and theoretical underpinnings. Pharmacological interventions will be excluded. Intervention characteristics to be examined include availability, effectiveness, ease of delivery/application, preference, patient understanding, contextual relevance, type of health professional able to apply it, appropriateness, feasibility and adaptability for use in the sub-Saharan African setting**.** These characteristics are usually influenced by culture, participant factors, or the context in which the intervention was originally developed. Adaptability of the intervention refers to the ease of modifying aspects of the intervention to make it relevant to the target population, thereby ensuring that the purpose of the intervention is achieved. Any other information or characteristics that further describe the psychosocial interventions will be considered.

### Control

- Placebo
- Pharmacological intervention.
- Routine/standard fertility.

### Outcomes

#### Primary outcome.

Effectiveness of psychosocial intervention assessed in terms of:

- Sexual satisfaction, defined as the degree to which an individual is satisfied or happy with the sexual aspect of his or her relationship [75].
- Marital satisfaction, defined as an individual’s emotional and attitudinal state toward their own marriage relationship [76]
  - Marital intimacy, defined as personal romantic or emotional communication that requires knowledge and understanding of another person to express thoughts and feelings [77].
  - Infertility-related quality of Life of persons with infertility problems.

#### Secondary outcomes.

- Quality of life (QoL), defined by WHO as an individual’s perception of their position in life in the context of the culture and value systems in which they live and in relation to their goals, expectations, standards, and concerns [78].
- Common mental health problems experienced by persons having infertility problems, such as depression and anxiety, just to mention a few. We will only consider mental health problems if it was investigated together with any of the aforementioned primary outcomes, such as sexual satisfaction, marital satisfaction, and marital intimacy. Studies assessing psychosocial problems such as depression, anxiety, stress, self-esteem, etc., but did not investigate sexual satisfaction, marital intimacy or QoL will be excluded.
- Sleep quality, defined as “an individual’s self-satisfaction with all aspects of the sleep experience” [79].
- Psychometric characteristics or properties of the intervention assessed in terms of:
  - Feasibility in resource-limited settings, such as a country in LMICs context
  - Adaptability to other LMICs settings
  - Cost of the intervention
  - Expertise required to deliver the intervention, including whether the intervention can be delivered by non-specialists in primary care settings
  - Ease of delivery of intervention in resource-limited settings, including whether the intervention can be delivered in peripheral health facilities by non-specialist healthcare providers
  - Preference by the patients
  - Preference of intervention by healthcare providers

#### Adverse events.

We will collect information on all adverse events and categorize them into non-serious and serious events. Adverse advents in each category will be stratified by type, frequency, duration, and timing of occurrence of the adverse event.

- **Non‐serious adverse events**─ to be categorized as mild, moderate or severe, including:
  - Emotional distress in response to an unpleasant experience that arises from the effect of the psychosocial interventions or memory of a particular unpleasant experience during, or after, the psychosocial intervention [80].
  - Resistance─ where participants of a therapy exhibit unconscious defense mechanisms that hinder them from acknowledging and dealing with certain feelings, thoughts, or behaviours [81].
  - Denial─ exhibiting a defense mechanism to the psychological process of refusing to accept or acknowledge a painful reality elicited by or during the delivery of the intervention [82].
  - Dependence─ when a participant becomes mentally and/or emotionally reliant on the therapist [83].
- **Serious adverse events**─ will include any untoward occurrence or effect that at any dose: results in death; life‐threatening outcome; requires hospitalisation or prolongation of existing hospitalisation; results in persistent or significant disability or incapacity.

### Searches for the identification of studies

The following electronic databases will be searched: PubMed, SCOPUS, CINAHL Complete, LILACS, Cochrane CENTRAL and PsycINFO (EBSCO), and Google Scholar from 2000 to 31^st^ March 2025, without any language restriction, using all the relevant search terms and their synonyms, singular and plural forms, and spelling variations, together with the individual countries adapted from the ScHARR 2022 classification of LMICs. We structured the search around three key concepts: infertility, psychosocial interventions, and name of each LMIC, included a combination of both index terms (e.g., MeSH terms in PubMed) and free-text terms to capture relevant variations in terminology. We have incorporated Medical Subject Headings (MeSH) and other controlled vocabulary where applicable, alongside text words to enhance sensitivity and carefully combined terms using Boolean operators (AND, OR) to ensure both breadth and precision. The revised strategy has been calibrated by running test searches to ensure it will retrieve all relevant studies”. The search strategy for PubMed has been reported in Table 1 and it will be adapted for use in the other databases. We will also search HINARI, African Journals Online, Conference Proceedings, Preprints and Thesis Repositories. Reference lists of relevant studies including relevant systematic reviews will be searched to retrieve studies missed by our searches. Experts in the field will be contacted for completed but unpublished studies.

**Table 1:** Search strategy for PubMed (to be adapted for the other databases).

Search	Query	Results
#1 Infertility	((Infertility[Text Word] OR childlessness[Text Word] OR subfertility[Text Word] OR “sub-fertility”[Text Word] OR sterility[Text Word] OR “reproductive sterility”[Text Word] OR infertile[Text Word] OR “male infertility”[Text Word] OR “female infertility”[Text Word] OR “infertile man”[Text Word] OR “infertile woman”[Text Word] OR “infertile couple”[Text Word] OR “primary infertility”[Text Word] OR “secondary infertility”[Text Word] OR “fertility issues”[Text Word] OR “fertility problems”[Text Word] OR “childless couple”[Text Word] OR “childless woman”[Text Word] OR barren[Text Word] OR barrenness[Text Word]) OR (“assisted reproductive techniques”[Text Word] OR ART[Text Word] OR “in vitro fertilization”[Text Word] OR IVF[Text Word] OR “intracytoplasmic sperm injection”[Text Word] OR ICSI[Text Word] OR “artificial insemination”[Text Word] OR “ovulation induction”[Text Word] OR “ovulation therapy”[Text Word])) OR (Infertility[MeSH Terms])	
#2 Psychosocial intervention	(((((“psychosocial intervention”[Text Word] OR “psychosocial education”[Text Word] OR “psychological support”[Text Word] OR psychotherapy[Text Word] OR counseling[Text Word] OR “cognitive behavioral therapy”[Text Word] OR CBT[Text Word] OR “educational intervention”[Text Word] OR “acceptance and commitment therapy”[Text Word] OR ACT[Text Word] OR “sex therapy”[Text Word] OR “sex counseling”[Text Word] OR “marital therapy”[Text Word] OR relaxation[Text Word] OR “directive counseling”[Text Word]) OR (psychosocial intervention[MeSH Terms])) OR (acceptance and commitment therapy[MeSH Terms])) OR (counseling[MeSH Terms])) OR “directive counseling”[Text Word]) OR (psychosocial intervention[MeSH Terms])) OR (acceptance and commitment therapy[MeSH Terms])) OR (counseling[MeSH Terms])) OR (directive counseling[MeSH Terms])) OR (sex counseling[MeSH Terms])	
#3 Country	Afghanistan[Text Word] OR albania[Text Word] OR algeria[Text Word] OR “american samoa”[Text Word] OR angola[Text Word] OR “antigua and barbuda”[Text Word] OR antigua[Text Word] OR barbuda[Text Word] OR argentina[Text Word] OR armenia[Text Word] OR armenian[Text Word] OR aruba[Text Word] OR azerbaijan[Text Word] OR bahrain[Text Word] OR bangladesh[Text Word] OR barbados[Text Word] OR republic of belarus[Text Word] OR belarus[Text Word] OR byelarus[Text Word] OR belorussia[Text Word] OR byelorussian[Text Word] OR belize[Text Word] OR “british Honduras”[Text Word] OR benin[Text Word] OR dahomey[Text Word] OR bhutan[Text Word] OR bolivia[Text Word] OR “bosnia and herzegovina”[Text Word] OR bosnia[Text Word] OR herzegovina[Text Word] OR botswana[Text Word] OR bechuanaland[Text Word] OR brazil[Text Word] OR brasil[Text Word] OR bulgaria[Text Word] OR “burkina faso”[Text Word] OR “burkina fasso”[Text Word] OR “upper volta”[Text Word] OR burundi[Text Word] OR urundi[Text Word] OR “cabo verde”[Text Word] OR “cape verde”[Text Word] OR cambodia[Text Word] OR kampuchea[Text Word] OR “khmer republic”[Text Word] OR cameroon[Text Word] OR cameron[Text Word] OR cameroun[Text Word] OR “central african republic”[Text Word] OR “ubangi shari”[Text Word] OR chad[Text Word] OR chile[Text Word] OR china[Text Word] OR colombia[Text Word] OR comoros[Text Word] OR “comoro islands”[Text Word] OR iles comores[Text Word] OR mayotte[Text Word] OR “democratic republic of the congo”[Text Word] OR “democratic republic congo”[Text Word] OR congo[Text Word] OR zaire[Text Word] OR costa rica[Text Word] OR “cote d’ivoire”[Text Word] OR “cote d’ ivoire”[Text Word] OR “cote d’ivoire”[Text Word] OR “cote d ivoire”[Text Word] OR “ivory coast”[Text Word] OR croatia[Text Word] OR cuba[Text Word] OR cyprus[Text Word] OR “czech republic”[Text Word] OR czechoslovakia[Text Word] OR djibouti[Text Word] OR “french Somaliland”[Text Word] OR dominica[Text Word] OR “dominican republic”[Text Word] OR ecuador[Text Word] OR egypt[Text Word] OR “united arab republic”[Text Word] OR “el Salvador”[Text Word] OR “equatorial guinea”[Text Word] OR “spanish guinea”[Text Word] OR eritrea[Text Word] OR estonia[Text Word] OR eswatini[Text Word] OR swaziland[Text Word] OR ethiopia[Text Word] OR fiji[Text Word] OR gabon[Text Word] OR “gabonese republic”[Text Word] OR gambia[Text Word] OR “georgia (republic)”[Text Word] OR georgian[Text Word] OR ghana[Text Word] OR gold coast[Text Word] OR gibraltar[Text Word] OR greece[Text Word] OR grenada[Text Word] OR guam[Text Word] OR guatemala[Text Word] OR guinea[Text Word] OR “guinea Bissau”[Text Word] OR guyana[Text Word] OR “british Guiana”[Text Word] OR haiti[Text Word] OR hispaniola[Text Word] OR honduras[Text Word] OR hungary[Text Word] OR india[Text Word] OR indonesia[Text Word] OR timor[Text Word] OR iran[Text Word] OR iraq[Text Word] OR isle of man[Text Word] OR jamaica[Text Word] OR jordan[Text Word] OR kazakhstan[Text Word] OR kazakh[Text Word] OR kenya[Text Word] OR “democratic people’s republic of korea”[Text Word] OR republic of korea[Text Word] OR “north korea”[Text Word] OR “south korea”[Text Word] OR korea[Text Word] OR kosovo[Text Word] OR kyrgyzstan[Text Word] OR kirghizia[Text Word] OR kirgizstan[Text Word] OR “kyrgyz republic”[Text Word] OR kirghiz[Text Word] OR laos[Text Word] OR “lao pdr”[Text Word] OR “lao people’s democratic republic”[Text Word] OR latvia[Text Word] OR lebanon[Text Word] OR “lebanese republic”[Text Word] OR lesotho[Text Word] OR basutoland[Text Word] OR liberia[Text Word] OR libya[Text Word] OR “libyan arab Jamahiriya”[Text Word] OR lithuania[Text Word] OR macau[Text Word] OR macao[Text Word] OR “republic of north Macedonia”[Text Word] OR macedonia[Text Word] OR madagascar[Text Word] OR “malagasy republic”[Text Word] OR malawi[Text Word] OR nyasaland[Text Word] OR malaysia[Text Word] OR “federated malay states”[Text Word] OR “federation of malaya”[Text Word] OR maldives[Text Word] OR “indian ocean islands”[Text Word] OR “indian ocean”[Text Word] OR mali[Text Word] OR malta[Text Word] OR micronesia[Text Word] OR “federated states of Micronesia”[Text Word] OR kiribati[Text Word] OR “marshall islands”[Text Word] OR nauru[Text Word] OR “northern mariana islands”[Text Word] OR palau[Text Word] OR tuvalu[Text Word] OR Mauritania[Text Word] OR mauritius[Text Word] OR mexico[Text Word] OR moldova[Text Word] OR moldovian[Text Word] OR mongolia[Text Word] OR	
	montenegro[Text Word] OR morocco[Text Word] OR ifni[Text Word] OR mozambique[Text Word] OR “portuguese east Africa”[Text Word] OR myanmar[Text Word] OR burma[Text Word] OR namibia[Text Word] OR nepal[Text Word] OR “netherlands Antilles”[Text Word] OR nicaragua[Text Word] OR niger[Text Word] OR nigeria[Text Word] OR oman[Text Word] OR muscat[Text Word] OR pakistan[Text Word] OR panama[Text Word] OR “papua new guinea”[Text Word] OR “new guinea”[Text Word] OR paraguay[Text Word] OR peru[Text Word] OR philippines[Text Word] OR philipines[Text Word] OR phillipines[Text Word] OR phillippines[Text Word] OR poland[Text Word] OR “polish people’s republic”[Text Word] OR portugal[Text Word] OR “portuguese republic”[Text Word] OR “puerto rico”[Text Word] OR romania[Text Word] OR russia[Text Word] OR “russian federation”[Text Word] OR ussr[Text Word] OR “soviet union”[Text Word] OR “union of soviet socialist republics”[Text Word] OR rwanda[Text Word] OR ruanda[Text Word] OR samoa[Text Word] OR “pacific islands”[Text Word] OR polynesia[Text Word] OR “samoan islands”[Text Word] OR navigator island[Text Word] OR navigator islands[Text Word] OR “sao tome and principe”[Text Word] OR “saudi arabia”[Text Word] OR senegal[Text Word] OR serbia[Text Word] OR seychelles[Text Word] OR “sierra leone”[Text Word] OR slovakia[Text Word] OR “slovak republic”[Text Word] OR slovenia[Text Word] OR melanesia[Text Word] OR “solomon island”[Text Word] OR “solomon islands”[Text Word] OR norfolk island[Text Word] OR norfolk islands[Text Word] OR somalia[Text Word] OR “south Africa”[Text Word] OR “south sudan”[Text Word] OR “sri lanka”[Text Word] OR ceylon[Text Word] OR “saint kitts and nevis”[Text Word] OR “st. kitts and nevis”[Text Word] OR saint lucia[Text Word] OR “st. lucia”[Text Word] OR “saint vincent and the grenadines”[Text Word] OR “saint Vincent”[Text Word] OR “st. vincent”[Text Word] OR grenadines[Text Word] OR sudan[Text Word] OR suriname[Text Word] OR surinam[Text Word] OR “dutch Guiana”[Text Word] OR netherlands Guiana[Text Word] OR syria[Text Word] OR “syrian arab republic”[Text Word] OR tajikistan[Text Word] OR tadjikistan[Text Word] OR tadzhikistan[Text Word] OR tadzhik[Text Word] OR tanzania[Text Word] OR tanganyika[Text Word] OR thailand[Text Word] OR siam[Text Word] OR “timor leste”[Text Word] OR “east timor”[Text Word] OR togo[Text Word] OR “togolese republic”[Text Word] OR tonga[Text Word] OR “trinidad and tobago”[Text Word] OR trinidad[Text Word] OR tobago[Text Word] OR tunisia[Text Word] OR turkey[Text Word] OR turkmenistan[Text Word] OR turkmen[Text Word] OR uganda[Text Word] OR ukraine[Text Word] OR uruguay[Text Word] OR uzbekistan[Text Word] OR uzbek[Text Word] OR vanuatu[Text Word] OR “new Hebrides”[Text Word] OR venezuela[Text Word] OR vietnam[Text Word] OR “viet nam”[Text Word] OR “middle east”[Text Word] OR “west bank”[Text Word] OR gaza[Text Word] OR palestine[Text Word] OR yemen[Text Word] OR yugoslavia[Text Word] OR zambia[Text Word] OR zimbabwe[Text Word] OR “northern Rhodesia”[Text Word] OR “global south”[Text Word] OR “africa south of the sahara”[Text Word] OR “sub-saharan Africa”[Text Word] OR “subsaharan Africa”[Text Word] OR “africa, central”[Text Word] OR “central Africa”[Text Word] OR “africa, northern”[Text Word] OR “north Africa”[Text Word] OR “northern Africa”[Text Word] OR magreb[Text Word] OR maghrib[Text Word] OR sahara[Text Word] OR “africa, southern”[Text Word] OR “southern Africa”[Text Word] OR “africa, eastern”[Text Word] OR “east Africa”[Text Word] OR “eastern Africa”[Text Word] OR “africa, western”[Text Word] OR “west Africa”[Text Word] OR “western Africa”[Text Word] OR “west indies”[Text Word] OR “indian ocean islands”[Text Word] OR caribbean[Text Word] OR “central America”[Text Word] OR “latin America”[Text Word] OR “south and central america”[Text Word] OR “south America”[Text Word] OR “asia, central”[Text Word] OR “central asia”[Text Word] OR “asia, northern”[Text Word] OR “north asia”[Text Word] OR “northern asia”[Text Word] OR “asia, southeastern”[Text Word] OR “southeastern asia”[Text Word] OR “south eastern asia”[Text Word] OR “southeast asia”[Text Word] OR “south east asia”[Text Word] OR “asia, western”[Text Word] OR “western asia”[Text Word] OR “europe, eastern”[Text Word] OR “east Europe”[Text Word] OR “eastern Europe”[Text Word]	
#4	#1 AND #2 AND #3	

### Managing the search results and selecting studies

**Fig 1:**
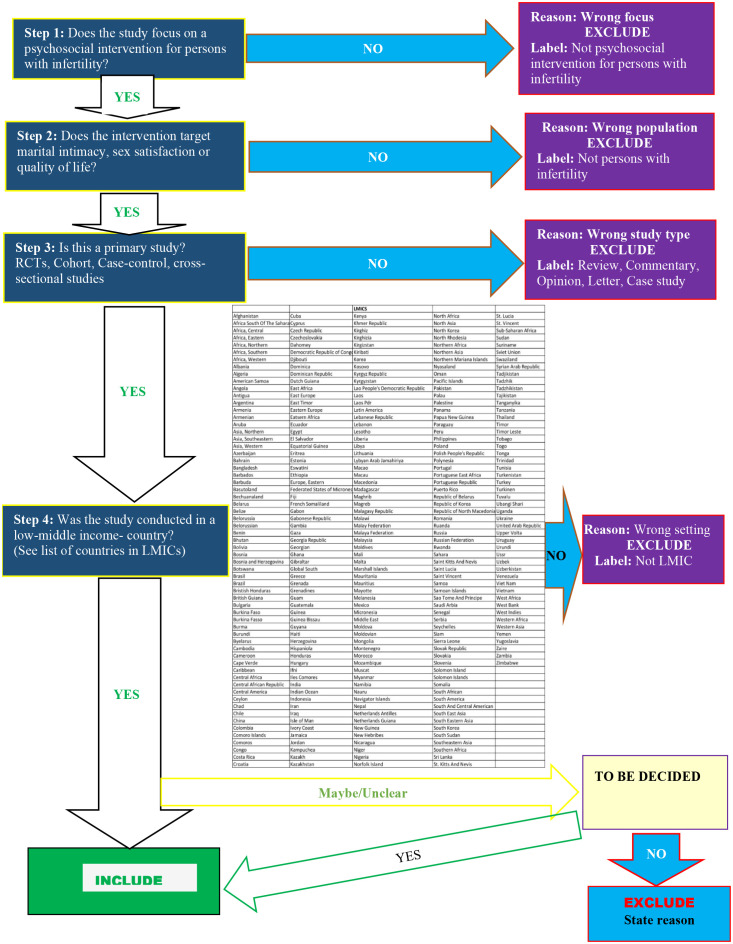
Study selection flow chart.

All studies retrieved from the various sources will be documented in a table and exported to Rayyan for de-duplication and study selection. A pre-tested study selection flow chart developed from the inclusion/exclusion criteria (Fig 1) will be used for study selection. At least two reviewers will screen titles and abstracts against the study selection flowchart to select all potentially relevant studies. Full documents or texts will be sought for these studies for final assessment and selection against our pre-specified eligibility criteria. Studies that meet all the pre-specified inclusion criteria will be included in the systematic review. Any disagreements between reviewers will be resolved through discussion.

### Data extraction and management

Data will be extracted using pretested data extraction form (S2 Table). The following information will be extracted: characteristics of the study including Study ID, country the study was conducted, year the study was conducted, sample size and setting (urban/rural); population variables such as age of participants, sex; type of psychosocial intervention (Supportive Program, Counselling, CBT, Educational Intervention, ACT and Relaxation Technique) all intervention characteristics such as format, intensity, frequency, number of sessions, duration of session, delivery mode, setting, provider qualifications, theoretical underpinnings and follow-up time; outcomes data (number achieving the desired effect of marital intimacy, sexual satisfaction, marital satisfaction, sleep quality as well as information on psychometric characteristics of the intervention such as cost, adaptability, feasibility, expertise required to deliver and intervention preference); and information on adverse events including type, frequency, duration, and timing of the occurrence of the adverse event. All data conversions will be made as part of the data extraction process before the data analysis. Any discrepancies between the data extractors will be discussed and resolved by consensus.

### Assessment of quality of the included studies for risk of bias

The Cochrane risk-of-bias tool for randomized trials (RoB 2) (S3 Table) will be used to assess risk of bias of included RCTs in five main domains: 1) risk of bias arising from the randomization process, 2) risk of bias due to deviations from the intended interventions (effect of assignment to intervention) and risk of bias due to deviations from the intended interventions (effect of adhering to intervention), 3) missing outcome data, 4) risk of bias in measurement of the outcome, and 5) risk of bias in selection of the reported result. Each of the domains has a number of signalling questions with responses to each signalling questions being ‘Yes (N)’, ‘Probably Yes (PY)’, ‘Probably No (PN)’, ‘No (N)’, and ‘No Information (NI)’. The risk of bias in each trial will be judged as ‘Low’, ‘High’ or ‘Some concerns’. The results from the risk of bias assessment will be presented in a table with supporting statements from the primary studies. The risk of bias in non-randomised studies – of interventions will be assessed using ROBINS-Iv2 tool from seven domains: 1) risk of bias due to confounding, 2) risk of bias in classification of interventions, 3) risk of bias in selection of participants into the study (or into the analysis), 4) risk of bias due to deviations from intended interventions, 5) risk of bias due to missing data, 6) risk of bias arising from measurement of the outcome and 7) risk of bias in selection of the reported result. Each bias domain in ROBINS-I is addressed using a series of signalling questions to gather important information about the study and the analysis being assessed. Most signalling questions have response options ‘Yes’, ‘Probably yes’, ‘Probably no’, ‘No’ and ‘No information’, with ‘Yes’ and ‘Probably yes’ having the same implications for risk of bias and similarly for ‘No’ and ‘Probably no’. Some questions have additional response options (a ‘weak’ and a ‘strong’ version of ‘Yes’ or ‘No’) to help discriminate between higher and lower risk of bias. The risk of bias in each trial is judged as *Low risk of bias* (there is little or no concern about bias with regard to this domain), *Moderate risk of bias* (there is some concern about bias with regard to this domain, although it is not clear that there is an important risk of bias, *Serious risk of bias* (the study has some important problems in this domain: characteristics of the study give rise to a serious risk of bias) or *Critical risk of bias* (the study is very problematic in this domain: characteristics of the study give rise to a critical risk of bias, such that and the result should generally be excluded from evidence syntheses). The quality of observational studies will be assessed using Hoy’s quality assessment tool (S4 Table) in four risk of bias domains: selection, non-response, measurement, and data analysis. For each domain, two independent reviewers will evaluate and classify it as having a ‘low risk of bias’, ‘high risk of bias’, or ‘unclear risk of bias’. The overall quality of evidence will be classified as low, high, or unclear risk of bias based on each study. Discrepancies will be resolved through discussion between the reviewers.

### Data analysis

The included studies will be summarized by their PICOS elements, including participant characteristics, study type, psychosocial interventions, and outcomes of interest. Review Manager v5.4 (and where necessary Stata version 18.0) will be used to analyze quantitative data. Measures of effect; Odds ratio (OR) or risk ratio (RR) will be utilized to present dichotomous data, while mean difference (MD) will be used to express continuous outcome data. Continuous outcomes data measured using different scales or instruments will be reported as standardized mean difference (SMD). All measures will be accompanied by their corresponding confidence intervals (CIs). The study-specific estimates will be used to determine standard deviations, where applicable, based on point estimates and the appropriate denominators, assuming a binomial distribution to assess heterogeneity between studies. Then the magnitude of heterogeneity between included studies will be assessed quantitatively using the index of heterogeneity (I^2^ statistics). The range of I^2^ values that will be considered for categorizing heterogeneity are: 25% for low, 50% for moderate, and ≥75% for significant heterogeneity. If the data from multi-country studies cannot be disaggregated because the results were initially pooled, these studies will be excluded from the meta-analysis.

Adverse advents will be categorized as non-serious or serious events. Given the lack of uniformity, and the fact that adverse events are usually poorly reported, we anticipate synthesizing adverse events outcome data narratively and reporting the results in tables, stratified by type of intervention, and providing clear description of patterns, context, frequency, duration, and timing of occurrence of the events. However, if data permit and the studies are sufficiently similar, we will compare event rates between intervention and control groups in meta-analysis and expressed as RR, OR, or risk difference (RD) using random-effects model. If the adverse events are rare, we will employ Peto odds ratio to estimate pooled event rates between the intervention and control. All estimates of adverse events will be reported with their 95% CIs.

### Heterogeneity and subgroup analysis

We will determine the significance of heterogeneity using the p-value of the I^2^ statistic, and a p-value <0.05 will be considered evidence of heterogeneity. Subgroup analysis will be performed where heterogeneity detected is significantly high and the possible sources will be explored. Heterogeneity will be assessed around variables such as sex, age of participants, study setting (hospital or community-based), geographical location (regions and subregions) and type of infertility. Sub-group analysis for RCTs will be done carefully in order not to break the randomization code of the trials.

### Sensitivity analysis

This is a process of testing the robustness of the results. Data will be re-analysed to determine if the results are sensitive to specific review elements [84]. The domains to be considered are the quality of the included studies, sample size and meta-analysis technique applied.

### Handling missing and incomplete data

An attempt will be made to extract sufficient data from all included studies. Where data on some pertinent variables are missing, the original authors of the primary studies will be contacted to see if they can provide the missing data. Where original authors are unable to provide the requested information or cannot be reached, we will not compute. Instead, the potential impact of missing data on the findings of the review will be addressed in the discussion section.

### Grading level of evidence

The Grading of Recommendations, Assessment, Development, and Evaluations (GRADE) approach will be used to assess the overall quality of evidence generated from the review for each outcome. The pooled data of each study will be evaluated against the five GRADE considered; risk of bias, imprecision, inconsistency, indirectness, and publication bias, and will be graded as high, high, moderate, low, and very low levels of evidence accordingly [85,86]. High-quality evidence is interpreted as further research is very unlikely to change the confidence in the estimate of effect, while grading of very low quality implies an estimate of effect is very doubtful.

### Ethics and dissemination

This study includes data from existing studies and does not require ethical approval. The findings of this systematic review will be made available to stakeholders, practitioners, patients and policy makers through presentation at scientific conferences and symposia. The review will be submitted for publication in peer-reviewed journal.

## Discussion

The impact of psychosocial stressors on the functional outcomes and overall QoL of infertile couples makes it an issue of public health concern, thus necessitating the inclusion of psychosocial intervention as a complementary management option of infertility [10]. This review aims to summarise the evidence of psychosocial interventions for improving marital intimacy, marital satisfaction, sexual satisfaction, and overall fertility-related QoL in LMICs and to provide the psychometric characteristics of the interventions within the regions and countries to ascertain their effectiveness and adaptability. The findings from the review will inform the choice and implementation that is context relevant and culturally friendly for infertility care. The use of a predefined review process and rigorously assessing risk of bias in the included studies will minimize bias and improve internal validity whereas the use of PICOS to define the population, intervention/exposure, control/comparator, outcomes and study type will ensure the internally valid reviews findings are also generalisable to the wider source population. Furthermore, given that the review questions and outcome measures have been developed in collaboration with the relevant patient, consumer and stakeholder involvement, the findings will inform their priorities, experiences.

This systematic review protocol has been developed with consideration of infertility as an integral component of sexual and reproductive health and rights, and which attempts to target the negative psychosocial impact in an effort towards achieving the SDG 3 which aims to ensure healthy lives and well-being for everyone at all ages, and SDG 5, which seeks to attain gender equality and empower all women and girls. In this context, the review will presents a comprehensive report to highlight the available psychosocial interventions and offer patient-led recommendations.

### Strengths and limitations

The study will report the first comprehensive systematic review and meta-analysis in LMICs to identify available psychosocial interventions and determine their impact on infertile couples, with particular emphasis on marital intimacy, sexual satisfaction and quality of life. In order to minimize bias in the review process, we will employ a very comprehensive search strategy to retrieve studies from all relevant database and non-database sources, and select studies, extract data and assess quality in the included studies independently using validated tools. The review plans to assess certainty of the overall (pooled) evidence using GRADE. A potential limitation is the anticipated diversity of psychosocial interventions that will make direct comparisons difficult. However, we will classify the types of interventions used in primary studies carefully and perform subgroup analyses, where necessary possible, to explore the source of the heterogeneity and the subgroup that will benefit most from the interventions. We acknowledge that some of the primary studies will be of low quality, we will perform sensitivity analysis on the quality of studies domain to test the robustness of our pooled evidence.

### Implications of the anticipated study findings

The findings from the proposed systematic review are expected to document psychosocial interventions in LMICs that improve marital intimacy and sexual satisfaction, ultimately impacting on the quality of life of people with infertility, particularly women. The findings will also identify knowledge gaps to inform future research and provide evidence-based solutions to improve psychosocial health care for people with infertility in LMICs.

## Supporting information

S1 Fig PRISMA Flow Diagram. (PDF)

S1 Table PRISMA-P guidelines. (PDF)

S2 Table Data extraction form. (PDF)

S3 Table Cochrane risk-of-bias tool for randomized trials (RoB 2). (PDF)

S4 Table Quality assessment tool for observational/prevalence studies. (PDF)

## References

[1] Starc A, Trampuš M, Pavan Jukić D, Rotim C, Jukić T, Polona Mivšek A. Infertility and sexual dysfunctions: a systematic literature review. Acta Clin Croat. 2019;58(3):508–15. doi: 10.20471/acc.2019.58.03.15 31969764 PMC6971809

[2] Zayed AA, El-Hadidy MA. Sexual satisfaction and self-esteem in women with primary infertility. Middle East Fertil Soc J. 2020;25(1). doi: 10.1186/s43043-020-00024-5

[3] Shayesteh-Parto F, Hasanpoor-Azghady SB, Arefi S, Amiri-Farahani L. Infertility-related stress and its relationship with emotional divorce among Iranian infertile people. BMC Psychiatry. 2023;23(1):666. doi: 10.1186/s12888-023-05159-z 37700231 PMC10496378

[4] Walker MH, Tobler KJ. Female Infertility. In: *StatPearls*. Treasure Island (FL): StatPearls Publishing. 2024. http://www.ncbi.nlm.nih.gov/books/NBK556033/32310493

[5] Sharma A, Shrivastava D. Psychological problems related to infertility. Cureus. 2022;14:e30320. 10.7759/cureus.30320 PMC9661871 36407201

[6] Kamboj N, Saraswathy KN, Prasad S, Babu N, Puri M, Sharma A, et al. Women infertility and common mental disorders: A cross-sectional study from North India. PLoS One. 2023;18(1):e0280054. doi: 10.1371/journal.pone.0280054 36603005 PMC9815660

[7] Kiani Z, Fakari FR, Hakimzadeh A, Hajian S, Fakari FR, Nasiri M. Prevalence of depression in infertile men: a systematic review and meta-analysis. BMC Public Health. 2023;23(1):1972. doi: 10.1186/s12889-023-16865-4 37821902 PMC10568846

[8] Khalesi ZB, Kenarsari FJ. Anxiety, depression, and stress: a comparative study between couples with male and female infertility. BMC Womens Health. 2024;24(1):228. doi: 10.1186/s12905-024-03072-5 38589804 PMC11003146

[9] Azize Diallo A, Anku PJ, Darkoa Oduro RA. Exploring the psycho-social burden of infertility: Perspectives of infertile couples in Cape Coast, Ghana. PLoS One. 2024;19(1):e0297428. doi: 10.1371/journal.pone.0297428 38271436 PMC10810504

[10] Azize Diallo A, Anku PJ, Darkoa Oduro RA. Exploring the psycho-social burden of infertility: Perspectives of infertile couples in Cape Coast, Ghana. PLoS One. 2024;19(1):e0297428. doi: 10.1371/journal.pone.0297428 38271436 PMC10810504

[11] Oppong SS, Naab F, Akuffo RA, Donkor ES. Functional status and quality of life of women with infertility in Southern Ghana: A cross-sectional study. HSI Journal. 2023;(Volume 4 Issue 2):550–9. doi: 10.46829/hsijournal.2023.12.4.2.550-559

[12] Dourou P, Gourounti K, Lykeridou A, Gaitanou K, Petrogiannis N, Sarantaki A. Quality of Life among Couples with a Fertility Related Diagnosis. Clin Pract. 2023;13(1):251–63. doi: 10.3390/clinpract13010023 36826165 PMC9955447

[13] Liu K, Dou S, Qin W, Zhao D, Zheng W, Wang D, et al. Association between quality of life and resilience in infertile patients: a systematic review. Front Public Health. 2024;12:1345899. doi: 10.3389/fpubh.2024.1345899 38476488 PMC10927801

[14] Hasin DS, Sarvet AL, Meyers JL, Saha TD, Ruan WJ, Stohl M, et al. Epidemiology of Adult DSM-5 Major Depressive Disorder and Its Specifiers in the United States. JAMA Psychiatry. 2018;75(4):336–46. doi: 10.1001/jamapsychiatry.2017.4602 29450462 PMC5875313

[15] Wang Y, Fu Y, Ghazi P, Gao Q, Tian T, Kong F, et al. Prevalence of intimate partner violence against infertile women in low-income and middle-income countries: a systematic review and meta-analysis. Lancet Glob Health. 2022;10(6):e820–30. doi: 10.1016/S2214-109X(22)00098-5 35561719 PMC9115867

[16] Heredia A, Padilla F, Castilla JA, Garcia-Retamero R. Effectiveness of a psychological intervention focused on stress management for women prior to IVF. J Reprod Infant Psychol. 2020;38(2):113–26. doi: 10.1080/02646838.2019.1601170 30990057

[17] Naab F, Brown R, Ward EC. Culturally adapted depression intervention to manage depression among women with infertility in Ghana. J Health Psychol. 2019. doi: 1359105319857175 10.1177/1359105319857175 31216898

[18] Tahere RF, Kalantarkousheh SM, Faramarzi M. The effect of mindfulness-based cognitive psychotherapy on quality of life in infertile women. Hayat. 2017;23:277–89.

[19] Asazawa K, Jitsuzaki M, Mori A, Ichikawa T. Effectiveness of a web-based partnership support program for preventing decline in the quality of life of male patients undergoing infertility treatment: A quasi-experimental study. Jpn J Nurs Sci. 2023;20(3):e12536. doi: 10.1111/jjns.12536 37057602

[20] Chan CHY, Lau BHP, Wong QS, Tam MYJ, So GYK, Leung HT, et al. Comparing the effectiveness of I-BMS-informed self-help interventions in alleviating psychosocial distress for women awaiting the outcome of IVF treatment. Asia Pacific Journal of Social Work and Development. 2019;29(3):179–93. doi: 10.1080/02185385.2019.1578684

[21] Kremer F, Ditzen B, Wischmann T. Effectiveness of psychosocial interventions for infertile women: A systematic review and meta-analysis with a focus on a method-critical evaluation. PLoS One. 2023;18(2):e0282065. doi: 10.1371/journal.pone.0282065 36854039 PMC9974119

[22] Withers M. Infertility among women in low- and middle-income countries. In: Kickbusch I, Ganten D, Moeti MS. Handbook of global health. Cham: Springer International Publishing. 2023. 885–910.

[23] Chiware TM, Vermeulen N, Blondeel K, Farquharson R, Kiarie J, Lundin K, et al. IVF and other ART in low- and middle-income countries: a systematic landscape analysis. Hum Reprod Update. 2021;27(2):213–28. doi: 10.1093/humupd/dmaa047 33238297 PMC7903111

[24] Connolly MP, Hoorens S, Chambers GM, ESHRE Reproduction and Society Task Force. The costs and consequences of assisted reproductive technology: an economic perspective. Hum Reprod Update. 2010;16(6):603–13. doi: 10.1093/humupd/dmq013 20530804

[25] Adhikary P, Mburu G, Kabra R, Habib NA, Kiarie J, Dhabhai N, et al. Intersectional analysis of the experiences of women who fail to conceive in low and middle income neighbourhoods of Delhi, India: Findings from a qualitative study. PLoS One. 2024;19(7):e0304029. doi: 10.1371/journal.pone.0304029 38959201 PMC11221677

[26] Kuug AK, James S, Sihaam J-B. Exploring the cultural perspectives and implications of infertility among couples in the Talensi and Nabdam Districts of the upper east region of Ghana. Contracept Reprod Med. 2023;8(1):28. doi: 10.1186/s40834-023-00225-z 37076914 PMC10114423

[27] Ofosu-Budu D, Hanninen V. Living as an infertile woman: the case of southern and northern Ghana. Reprod Health. 2020;17(1):69. doi: 10.1186/s12978-020-00920-z 32434580 PMC7240982

[28] Thoma M, Fledderjohann J, Cox C, Kantum Adageba R. Biological and Social Aspects of Human Infertility: A Global Perspective. Oxford Research Encyclopedia of Global Public Health. Oxford University Press. 2021. doi: 10.1093/acrefore/9780190632366.013.184

[29] Koser K. Fertility Counseling With Couples: A Theoretical Approach. The Family Journal. 2020;28(1):25–32. doi: 10.1177/1066480719887498

[30] Chiware TM, Vermeulen N, Blondeel K, Farquharson R, Kiarie J, Lundin K, et al. IVF and other ART in low- and middle-income countries: a systematic landscape analysis. Hum Reprod Update. 2021;27(2):213–28. doi: 10.1093/humupd/dmaa047 33238297 PMC7903111

[31] Njagi P, Groot W, Arsenijevic J, Dyer S, Mburu G, Kiarie J. Financial costs of assisted reproductive technology for patients in low- and middle-income countries: a systematic review. Hum Reprod Open. 2023;2023(2):hoad007. doi: 10.1093/hropen/hoad007 36959890 PMC10029849

[32] Bayoumi RR, Koert E, Boivin J, Viswanath K, McConnell M. Quality of life of Sudanese patients attending a fertility clinic: a mixed methods study. Health Psychol Behav Med. 2021;9(1):1006–30. doi: 10.1080/21642850.2021.2007773 34881115 PMC8648023

[33] Pradhan Shrestha S, Bhandari SD, Pradhan S. Quality of Life among Infertile Women Attending an Infertility Treatment Center, Kathmandu. J Nepal Health Res Counc. 2020;18(3):394–400. doi: 10.33314/jnhrc.v18i3.2639 33210629

[34] Ombelet W, Onofre J. IVF in Africa: what is it all about?. Facts Views Vis Obgyn. 2019;11(1):65–76. 31695859 PMC6822948

[35] Ombelet W. WHO fact sheet on infertility gives hope to millions of infertile couples worldwide. Facts Views Vis Obgyn. 2020;12(4):249–51. 33575673 PMC7863696

[36] England MJ, Butler AS, Gonzalez ML. Psychosocial Interventions for Mental and Substance Use Disorders: A Framework for Establishing Evidence-Based Standards. Psychosocial Interventions for Mental and Substance Use Disorders: A Framework for Establishing Evidence-Based Standards. National Academies Press (US). 2015. 26203478

[37] Fatemeh FS, Khalatbari J, Rahmati S. Effectiveness of cognitive behavioral therapy based on body image on sexual satisfaction and marital adjustment of married infertile women. Applied Psychology. 2018;12:25–46.

[38] Marvi N, Golmakani N, Miri HH, Esmaily H. The Effect of Sexual Education based on Sexual Health Model on the Sexual Function of Women with Infertility. Iran J Nurs Midwifery Res. 2019;24(6):444–50. doi: 10.4103/ijnmr.IJNMR_199_17 31772919 PMC6875883

[39] Sax MR, Lawson AK. Emotional Support for Infertility Patients: Integrating Mental Health Professionals in the Fertility Care Team. Women. 2022;2(1):68–75. doi: 10.3390/women2010008

[40] Opland C, Torrico TJ. Psychodynamic Therapy. StatPearls. Treasure Island (FL): StatPearls Publishing. 2024. 39163451

[41] Prasko J, Ociskova M, Vanek J. Managing transference and countertransference in cognitive behavioral supervision: Theoretical framework and clinical application. Psychology Research and Behavior Management. 2022;15:2129. 35990755 10.2147/PRBM.S369294 PMC9384966

[42] Nakao M, Shirotsuki K, Sugaya N. Cognitive-behavioral therapy for management of mental health and stress-related disorders: Recent advances in techniques and technologies. Biopsychosoc Med. 2021;15(1):16. doi: 10.1186/s13030-021-00219-w 34602086 PMC8489050

[43] Derella OJ, Johnston OG, Loeber R, Burke JD. CBT-Enhanced Emotion Regulation as a Mechanism of Improvement for Childhood Irritability. J Clin Child Adolesc Psychol. 2019;48(sup1):S146–54. doi: 10.1080/15374416.2016.1270832 28151019 PMC6283701

[44] Greil AL, Slauson-Blevins K, McQuillan J, Lowry MH, Burch AR, Shreffler KM. Relationship Satisfaction Among Infertile Couples: Implications of Gender and Self-Identification. Journal of Family Issues. 2018;39(5):1304–25. doi: 10.1177/0192513x17699027

[45] Sahraian K, Abdollahpour Ranjbar H, Namavar Jahromi B, Cheung HN, Ciarrochi J, Habibi Asgarabad M. Effectiveness of mindful self-compassion therapy on psychopathology symptoms, psychological distress and life expectancy in infertile women treated with in vitro fertilization: a two-arm double-blind parallel randomized controlled trial. BMC Psychiatry. 2024;24(1):174. doi: 10.1186/s12888-023-05411-6 38429659 PMC10908010

[46] Sutton J. Humanistic Therapy: Unlocking Your Clients’ True Potential. PositivePsychology.com. 2024. https://positivepsychology.com/humanistic-therapy/

[47] Dimidjian S, Martell CR, Herman-Dunn R. Behavioral activation for depression. Clinical handbook of psychological disorders: A step-by-step treatment manual. 6th ed. New York, NY, US: The Guilford Press. 2021. 339–80.

[48] Kirca N, Pasinlioglu T. The effect of yoga on stress level in infertile women. Perspect Psychiatr Care. 2019;55(2):319–27. doi: 10.1111/ppc.12352 30657179

[49] Belevska J. The Impact of Psycho-Education on in Vitro Fertilisation Treatment Efficiency. Pril (Makedon Akad Nauk Umet Odd Med Nauki). 2015;36(2):211–6. doi: 10.1515/prilozi-2015-0069 27442387

[50] Javadivala Z, Allahverdipour H, Asghari Jafarabadi M, Azimi S, Gilani N, Chattu VK. Improved couple satisfaction and communication with marriage and relationship programs: are there gender differences?-a systematic review and meta-analysis. Syst Rev. 2021;10(1):178. doi: 10.1186/s13643-021-01719-0 34148550 PMC8215832

[51] Saxler E, Schindler T, Philipsen A, Schulze M, Lux S. Therapeutic alliance in individual adult psychotherapy: a systematic review of conceptualizations and measures for face-to-face- and online-psychotherapy. Front Psychol. 2024;15:1293851. doi: 10.3389/fpsyg.2024.1293851 38993343 PMC11238262

[52] Dailey J, Timulak L, Goldman RS, Greenberg LS. Capturing the change: a case study investigation of emotional and interactional transformation in emotion-focused therapy for couples. Person-Centered & Experiential Psychotherapies. 2023;23(1):1–19. doi: 10.1080/14779757.2023.2204480

[53] Ardelt M, Grunwald S. The Importance of Self-Reflection and Awareness for Human Development in Hard Times. Research in Human Development. 2018;15(3–4):187–99. doi: 10.1080/15427609.2018.1489098

[54] Gameiro S, Leone D, Mertes H. Fertility clinics have a duty of care towards patients who do not have children with treatment. Hum Reprod. 2024;39(8):1591–8. doi: 10.1093/humrep/deae128 38890127 PMC11291940

[55] Aiyenigba AO, Weeks AD, Rahman A. Managing Psychological Trauma of Infertility. Afr J Reprod Health. 2019;23(2):76–91. doi: 10.29063/ajrh2019/v23i2.8 31433596

[56] Armah D, van der Wath A, Yazbek M, Naab F. Holistic management of female infertility: A systematic review. Afr J Reprod Health. 2021;25(2):150–61. doi: 10.29063/ajrh2021/v25i2.15 37585763

[57] Brown RCH, Rogers WA, Entwistle VA, Bhattacharya S. Reframing the Debate Around State Responses to Infertility: Considering the Harms of Subfertility and Involuntary Childlessness. Public Health Ethics. 2016;9(3):290–300. doi: 10.1093/phe/phw005

[58] Chow K-M, Cheung M-C, Cheung IK. Psychosocial interventions for infertile couples: a critical review. Journal of Clinical Nursing. 2016;25:2101–13. 27278496 10.1111/jocn.13361

[59] Naab F, Brown R, Ward EC. Culturally adapted depression intervention to manage depression among women with infertility in Ghana. J Health Psychol. 2021;26(7):949–61. doi: 10.1177/1359105319857175 31216898

[60] Seyedi Asl ST, Sadeghi K, Bakhtiari M, Ahmadi SM, Nazari Anamagh A, Khayatan T. Effect of Group Positive Psychotherapy on Improvement of Life Satisfaction and The Quality of Life in Infertile Woman. Int J Fertil Steril. 2016;10(1):105–12. doi: 10.22074/ijfs.2016.4775 27123207 PMC4845519

[61] Zhou R, Cao Y-M, Liu D, Xiao J-S. Pregnancy or Psychological Outcomes of Psychotherapy Interventions for Infertility: A Meta-Analysis. Front Psychol. 2021;12:643395. doi: 10.3389/fpsyg.2021.643395 33868114 PMC8044306

[62] WHO. Infertility Prevalence Estimates, 1990–2021. 2023. https://www.who.int/publications-detail-redirect/978920068315

[63] Fang J, Tang S, Tan X, Tolhurst R. Achieving SDG related sexual and reproductive health targets in China: what are appropriate indicators and how we interpret them?. Reprod Health. 2020;17(1):84. doi: 10.1186/s12978-020-00924-9 32487257 PMC7268468

[64] Shah PK, Gher JM. Human rights approaches to reducing infertility. Int J Gynaecol Obstet. 2023;162(1):368–74. doi: 10.1002/ijgo.14878 37246463

[65] Perets S, Davidovich N, Lewin E. Perceptions of leadership, self-confidence and leadership programs among teenage girls in Israel. Cogent Education. 2023;10(1). doi: 10.1080/2331186x.2023.2195742

[66] Alirezaei S, Taghipour A, Latifnejad Roudsari R. The effect of infertility counseling interventions on marital and sexual satisfaction of infertile couples: A systematic review and meta-analysis. Int J Reprod Biomed. 2022;20(10):795–806. doi: 10.18502/ijrm.v20i10.12264 36381353 PMC9644646

[67] Ahmadnia E, Haseli A, Karamat A. Therapeutic interventions conducted on improving women’s sexual satisfaction and function during reproductive ages in Iran: A systematic review. Journal of Mazandaran University of Medical Sciences. 2017;27:146–62.

[68] Golshani F, Mirghafourvand M, Hasanpour S, Seiiedi Biarag L. The Effect of Cognitive Behavioral Therapy on Anxiety and Depression in Iranian Infertile Women: A Systematic and Meta-Analytical Review. Iran J Psychiatry Behav Sci. 2020;14(1). doi: 10.5812/ijpbs.96715

[69] WHO TEAM. Infertility Prevalence Estimates, 1990–2021. 2023. https://www.who.int/publications-detail-redirect/978920068315

[70] Deeks JJ, Bossuyt PM, Leeflang MM, Takwoingi Y. Cochrane Handbook for Systematic Reviews of Diagnostic Test Accuracy. Wiley. 2023. doi: 10.1002/9781119756194 PMC10408284 37470764

[71] Moher D, Shamseer L, Clarke M, Ghersi D, Liberati A, Petticrew M, et al. Preferred reporting items for systematic review and meta-analysis protocols (PRISMA-P) 2015 statement. Syst Rev. 2015;4(1):1. doi: 10.1186/2046-4053-4-1 25554246 PMC4320440

[72] Page MJ, McKenzie JE, Bossuyt PM, Boutron I, Hoffmann TC, Mulrow CD, et al. The PRISMA 2020 statement: an updated guideline for reporting systematic reviews. Syst Rev. 2021;10(1):89. doi: 10.1186/s13643-021-01626-4 33781348 PMC8008539

[73] Staniszewska S, Brett J, Simera I, Seers K, Mockford C, Goodlad S, et al. GRIPP2 reporting checklists: tools to improve reporting of patient and public involvement in research. BMJ. 2017;358:j3453. doi: 10.1136/bmj.j3453 28768629 PMC5539518

[74] WHO. Infertility. 2023. https://www.who.int/news-room/fact-sheets/detail/infertility

[75] Dienberg M-F, Oschatz T, Piemonte JL, Klein V. Women’s Orgasm and Its Relationship with Sexual Satisfaction and Well-being. Curr Sex Health Rep. 2023;15(3):223–30. doi: 10.1007/s11930-023-00371-0

[76] Abreu-Afonso J, Ramos MM, Queiroz-Garcia I, Leal I. How Couple’s Relationship Lasts Over Time? A Model for Marital Satisfaction. Psychol Rep. 2022;125(3):1601–27. doi: 10.1177/00332941211000651 33736540 PMC9136471

[77] Kardan-Souraki M, Hamzehgardeshi Z, Asadpour I, Mohammadpour RA, Khani S. A Review of Marital Intimacy-Enhancing Interventions among Married Individuals. Glob J Health Sci. 2016;8(8):53109. doi: 10.5539/gjhs.v8n8p74 27045400 PMC5016345

[78] WHOQOL-BREF: Measuring Quality of Life. 2020. https://www.who.int/tools/whoqol/whoqol-bref

[79] Nelson KL, Davis JE, Corbett CF. Sleep quality: An evolutionary concept analysis. Nurs Forum. 2022;57(1):144–51. doi: 10.1111/nuf.12659 34610163

[80] Wang Y, Tian J, Yang Q. Experiential Avoidance Process Model: A Review of the Mechanism for the Generation and Maintenance of Avoidance Behavior. Psychiatry Clin Psychopharmacol. 2024;34(2):179–90. doi: 10.5152/pcp.2024.23777 39165887 PMC11332439

[81] Di Giuseppe M, Perry JC. The Hierarchy of Defense Mechanisms: Assessing Defensive Functioning With the Defense Mechanisms Rating Scales Q-Sort. Front Psychol. 2021;12:718440. doi: 10.3389/fpsyg.2021.718440 34721167 PMC8555762

[82] Kumari S. Understanding defense mechanisms: How our minds protect us. International Journal of Research Culture Society. 2024;8(7). doi: 10.2017/IJRCS/202407024

[83] Perrotta G. Affective Dependence: From Pathological Affectivity to Personality Disorders: Definitions, Clinical Contexts, Neurobiological Profiles and Clinical Treatments. Health Sci. 2021;2(2021). doi: 10.15342/hs.2020.333

[84] Mowbray FI, Manlongat D, Shukla M. Sensitivity Analysis: A Method to Promote Certainty and Transparency in Nursing and Health Research. Can J Nurs Res. 2022;54(4):371–6. doi: 10.1177/08445621221107108 35702010 PMC9605992

[85] Granholm A, Alhazzani W, Møller MH. Use of the GRADE approach in systematic reviews and guidelines. Br J Anaesth. 2019;123(5):554–9. doi: 10.1016/j.bja.2019.08.015 31558313

[86] Zähringer J, Schwingshackl L, Movsisyan A, Stratil JM, Capacci S, Steinacker JM, et al. Use of the GRADE approach in health policymaking and evaluation: a scoping review of nutrition and physical activity policies. Implement Sci. 2020;15(1):37. doi: 10.1186/s13012-020-00984-2 32448231 PMC7245872

