# Supplementary material for: Psychosocial interventions for improving marital intimacy, sexual satisfaction, and quality of life of women and couples with infertility problems in low and middle-income countries: Systematic review protocol
Source: PLoS One. 2025 Oct 30;20(10):e0335068. doi: 10.1371/journal.pone.0335068 (PMC12574878; doi:10.1371/journal.pone.0335068)
Supplement: S2 Table — (PDF) [file pone.0335068.s003.pdf]

DATA EXTRACTION FORM*

Section A: Characteristics of the included studies			
**Variable**	**Information/data**	**Location in text where information was extracted**	**Notes**
Study ID*			
Country where study was conducted			
Year study was conducted			
Study design type			
Sample size			
Setting of participants (urban/rural)			
Health facility type			
Public/private facility?			
Age of participants (mean, median, range etc)			
Type of infertility			
Number of times infertility treatment sought			
Number of times patient became pregnant with infertility treatment			
Criteria for diagnosing infertility			
Period of screening for infertility			
Cut-off (if any applied to diagnostic tool)			
Psychosocial intervention received			
Format of delivery (individual or group)			
Number of sessions			
Duration of a session			
Duration of the full course of intervention			
Intervention delivered by (specialist/non-specialist etc)?			
Follow up time of the intervention			
Outcomes reported			
Any important remarks			
**Section B: Data on specific outcomes for the analysis**			
***Primary outcomes (effectiveness of intervention)*:**<br>Sexual satisfaction<br>Marital satisfaction<br>Marital intimacy<br>Infertility-related quality of life			
Secondary outcomes:<br>Sleep quality<br>Quality of life<br>Common mental health problems			
***Psychometric characteristics of interventions*:**<br>Feasibility<br>Adaptability<br>Expertise required<br>Cost of intervention			

<table>
<tr><td colspan="5">Ease of delivery Preference (by patients, health professionals etc)</td><td colspan="3"></td><td colspan="2"></td><td></td></tr>
<tr><td colspan="5">Adverse events
Non-serious (emotional distress, resistance, denial, dependence, etc)
Serious (death,
(categorized by frequency, duration, timing of occurrence etc)
Specific adverse events</td><td colspan="3"></td><td colspan="2"></td><td></td></tr>
<tr><td colspan="5"></td><td colspan="3"></td><td colspan="2"></td><td></td></tr>
<tr><td colspan="11">Section C: Data for the analyses</td></tr>
<tr><td colspan="5"></td><td colspan="3"></td><td colspan="2"></td><td></td></tr>
<tr><td colspan="11">Binary/dichotomous outcome (non-comparative)</td></tr>
<tr><td colspan="5">No. of events
(n)</td><td colspan="3">Sample size
(N)</td><td colspan="2"></td><td></td></tr>
<tr><td colspan="5"></td><td colspan="3"></td><td colspan="2"></td><td></td></tr>
<tr><td colspan="10">Binary/dichotomous outcome (comparative)</td><td></td></tr>
<tr><td colspan="2">No. of events
(n)</td><td colspan="2">Sample size (N)</td><td colspan="3">No. of events
(n)</td><td colspan="2">Sample size (N)</td><td></td><td></td></tr>
<tr><td colspan="2"></td><td colspan="2"></td><td colspan="3"></td><td colspan="2"></td><td></td><td></td></tr>
<tr><td colspan="9">Continuous outcome (non-comparative)</td><td></td><td></td></tr>
<tr><td colspan="3">Mean</td><td colspan="3">SD</td><td colspan="3">Sample size (N)</td><td></td><td></td></tr>
<tr><td colspan="3"></td><td colspan="3"></td><td colspan="3"></td><td></td><td></td></tr>
<tr><td colspan="9">Continuous outcome (comparative)</td><td></td><td></td></tr>
<tr><td>Mean</td><td colspan="2">SD</td><td>Sample size
(N)</td><td colspan="2">Mean</td><td>SD</td><td colspan="2">Sample size (N)</td><td></td><td></td></tr>
</table>

*This represents a row (for each study) in the very comprehensive Excel Spreadsheet is being developed. Each outcome or sub-categories will be presented as a separate column in the Excel spreadsheet. **Section A** will collect information about characteristics of the study and **Sections B and C** synthesis information (together with relevant demographic and intervention characteristics for each outcome (both narrative and quantitative). **Section C** will be used to extract data for quantitative analysis (including meta-analysis).
