# Supplementary material for: Psychosocial interventions for improving marital intimacy, sexual satisfaction, and quality of life of women and couples with infertility problems in low and middle-income countries: Systematic review protocol
Source: PLoS One. 2025 Oct 30;20(10):e0335068. doi: 10.1371/journal.pone.0335068 (PMC12574878; doi:10.1371/journal.pone.0335068)
Supplement: S3 Table — (PDF) [file pone.0335068.s004.pdf]

Revised Cochrane risk-of-bias tool for randomized trials (RoB 2)
TEMPLATE FOR COMPLETION

Edited by Julian PT Higgins, Jelena Savović, Matthew J Page, Jonathan AC Sterne
on behalf of the RoB2 Development Group

Version of 22 August 2019

The development of the RoB 2 tool was supported by the MRC Network of Hubs for Trials Methodology Research (MR/L004933/2- N61), with the support of the host MRC ConDuCT-II Hub (Collaboration and innovation for Difficult and Complex randomised controlled Trials In Invasive procedures - MR/K025643/1), by MRC research grant MR/M025209/1, and by a grant from The Cochrane Collaboration.

This work is licensed under a Creative Commons Attribution-NonCommercial-NoDerivatives 4.0 International License.

Study details

Reference

Study design

X Individually-randomized parallel-group trial
⬜ Cluster-randomized parallel-group trial
⬜ Individually randomized cross-over (or other matched) trial

For the purposes of this assessment, the interventions being compared are defined as

Experimental: Comparator:

Specify which outcome is being assessed for risk of bias

Specify the numerical result being assessed. In case of multiple alternative analyses being presented, specify the numeric result (e.g. RR = 1.52 (95% CI 0.83 to 2.77) and/or a reference (e.g. to a table, figure or paragraph) that uniquely defines the result being assessed.

Is the review team's aim for this result…?

⬜ to assess the effect of *assignment to intervention* (the 'intention-to-treat' effect)
⬜ to assess the effect of *adhering to intervention* (the 'per-protocol' effect)

If the aim is to assess the effect of *adhering to intervention*, select the deviations from intended intervention that should be addressed (at least one must be checked):

- ☐ occurrence of non-protocol interventions
- ☐ failures in implementing the intervention that could have affected the outcome
- ☐ non-adherence to their assigned intervention by trial participants

Which of the following sources were <u>obtained</u> to help inform the risk-of-bias assessment? (tick as many as apply)

- ☐ Journal article(s) with results of the trial
- ☐ Trial protocol
- ☐ Statistical analysis plan (SAP)
- ☐ Non-commercial trial registry record (e.g. ClinicalTrials.gov record)
- ☐ Company-owned trial registry record (e.g. GSK Clinical Study Register record)
- ☐ “Grey literature” (e.g. unpublished thesis)
- ☐ Conference abstract(s) about the trial
- ☐ Regulatory document (e.g. Clinical Study Report, Drug Approval Package)
- ☐ Research ethics application
- ☐ Grant database summary (e.g. NIH RePORTER or Research Councils UK Gateway to Research)
- ☐ Personal communication with trialist
- ☐ Personal communication with the sponsor

Risk of bias assessment

Responses underlined in green are potential markers for low risk of bias, and responses in red are potential markers for a risk of bias. Where questions relate only to sign posts to other questions, no formatting is used.

Domain 1: Risk of bias arising from the randomization process

Signalling questions	Comments	Response options
**1.1 Was the allocation sequence random?**		Y / PY / PN / N / NI
**1.2 Was the allocation sequence concealed until participants were enrolled and assigned to interventions?**		Y / PY / PN / N / NI
**1.3 Did baseline differences between intervention groups suggest a problem with the randomization process?**		Y / PY / PN / N / NI
**Risk-of-bias judgement**		Low / High / Some concerns
Optional: What is the predicted direction of bias arising from the randomization process?		NA / Favours experimental / Favours comparator / Towards null /Away from null / Unpredictable

Domain 2: Risk of bias due to deviations from the intended interventions (*effect of assignment to intervention*)

Signalling questions	Comments	Response options
**2.1. Were participants aware of their assigned intervention during the trial?**		Y / PY / PN / N / NI
**2.2. Were carers and people delivering the interventions aware of participants' assigned intervention during the trial?**		Y / PY / PN / N / NI
**2.3. If Y/PY/NI to 2.1 or 2.2: Were there deviations from the intended intervention that arose because of the trial context?**		NA / Y / PY / PN / N / NI
**2.4 If Y/PY to 2.3: Were these deviations likely to have affected the outcome?**		NA / Y / PY / PN / N / NI
**2.5. If Y/PY/NI to 2.4: Were these deviations from intended intervention balanced between groups?**		NA / Y / PY / PN / N / NI
**2.6 Was an appropriate analysis used to estimate the effect of assignment to intervention?**		Y / PY / PN / N / NI
**2.7 If N/PN/NI to 2.6: Was there potential for a substantial impact (on the result) of the failure to analyse participants in the group to which they were randomized?**		NA / Y / PY / PN / N / NI
**Risk-of-bias judgement**		Low / High / Some concerns
Optional: What is the predicted direction of bias due to deviations from intended interventions?		NA / Favours experimental / Favours comparator / Towards null /Away from null / Unpredictable

Domain 2: Risk of bias due to deviations from the intended interventions (*effect of adhering to intervention*)

Signalling questions	Comments	Response options
**2.1. Were participants aware of their assigned intervention during the trial?**		Y / PY / PN / N / NI
**2.2. Were carers and people delivering the interventions aware of participants' assigned intervention during the trial?**		Y / PY / PN / N / NI
**2.3. [If applicable:] If Y/PY/NI to 2.1 or 2.2: Were important non-protocol interventions balanced across intervention groups?**		NA / Y / PY / PN / N / NI
**2.4. [If applicable:] Were there failures in implementing the intervention that could have affected the outcome?**		NA / Y / PY / PN / N / NI
**2.5. [If applicable:] Was there non-adherence to the assigned intervention regimen that could have affected participants' outcomes?**		NA / Y / PY / PN / N / NI
**2.6. If N/PN/NI to 2.3, or Y/PY/NI to 2.4 or 2.5: Was an appropriate analysis used to estimate the effect of adhering to the intervention?**		NA / Y / PY / PN / N / NI
**Risk-of-bias judgement**		Low / High / Some concerns
Optional: What is the predicted direction of bias due to deviations from intended interventions?		NA / Favours experimental / Favours comparator / Towards null /Away from null / Unpredictable

Domain 3: Missing outcome data

Signalling questions	Comments	Response options
**3.1 Were data for this outcome available for all, or nearly all, participants randomized?**		Y / PY / PN / N / NI
**3.2 If N/PN/NI to 3.1: Is there evidence that the result was not biased by missing outcome data?**		NA / Y / PY / PN / N
**3.3 If N/PN to 3.2: Could missingness in the outcome depend on its true value?**		NA / Y / PY / PN / N / NI
**3.4 If Y/PY/NI to 3.3: Is it likely that missingness in the outcome depended on its true value?**		NA / Y / PY / PN / N / NI
**Risk-of-bias judgement**		Low / High / Some concerns
Optional: What is the predicted direction of bias due to missing outcome data?		NA / Favours experimental / Favours comparator / Towards null /Away from null / Unpredictable

Domain 4: Risk of bias in measurement of the outcome

Signalling questions	Comments	Response options
**4.1 Was the method of measuring the outcome inappropriate?**		Y / PY / PN / N / NI
**4.2 Could measurement or ascertainment of the outcome have differed between intervention groups?**		Y / PY / PN / N / NI
**4.3 If N/PN/NI to 4.1 and 4.2: Were outcome assessors aware of the intervention received by study participants?**		NA / Y / PY / PN / N / NI
**4.4 If Y/PY/NI to 4.3: Could assessment of the outcome have been influenced by knowledge of intervention received?**		NA / Y / PY / PN / N / NI
**4.5 If Y/PY/NI to 4.4: Is it likely that assessment of the outcome was influenced by knowledge of intervention received?**		NA / Y / PY / PN / N / NI
**Risk-of-bias judgement**		Low / High / Some concerns
Optional: What is the predicted direction of bias in measurement of the outcome?		NA / Favours experimental / Favours comparator / Towards null /Away from null / Unpredictable

Domain 5: Risk of bias in selection of the reported result

Signalling questions	Comments	Response options
**5.1 Were the data that produced this result analysed in accordance with a pre-specified analysis plan that was finalized before unblinded outcome data were available for analysis?**		Y / PY / PN / N / NI
**Is the numerical result being assessed likely to have been selected, on the basis of the results, from...**		
**5.2. ... multiple eligible outcome measurements (e.g. scales, definitions, time points) within the outcome domain?**		Y / PY / PN / N / NI
**5.3 ... multiple eligible analyses of the data?**		Y / PY / PN / N / NI
**Risk-of-bias judgement**		Low / High / Some concerns
Optional: What is the predicted direction of bias due to selection of the reported result?		NA / Favours experimental / Favours comparator / Towards null /Away from null / Unpredictable

Overall risk of bias

**Risk-of-bias judgement**		Low / High / Some concerns
Optional: What is the overall predicted direction of bias for this outcome?		NA / Favours experimental / Favours comparator / Towards null /Away from null / Unpredictable

This work is licensed under a Creative Commons Attribution-NonCommercial-NoDerivatives 4.0 International License.
